# Supplementary material for: A scoping review of ‘Pacing’ for management of Myalgic Encephalomyelitis/Chronic Fatigue Syndrome (ME/CFS): lessons learned for the long COVID pandemic
Source: J Transl Med. 2023 Oct 14;21:720. doi: 10.1186/s12967-023-04587-5 (PMC10576275; doi:10.1186/s12967-023-04587-5)
Supplement: Supplementary file 1 — Additional file 1. Supplementary file 1. Full search string for databse searching. [file 12967_2023_4587_MOESM1_ESM.docx]

**Appendix 1. Full Search Strings**

‘ME/CFS’ OR ‘ME’ OR ‘CFS’ OR ‘chronic fatigue syndrome’ OR ‘PEM’ OR ‘post exertional malaise’ OR ‘pene’ OR ‘post-exertion neurogenic exhaust’ AND ‘pacing’

‘ME/CFS’ OR ‘ME’ OR ‘CFS’ OR ‘chronic fatigue syndrome’ OR ‘PEM’ OR ‘post exertional malaise’ OR ‘pene’ OR ‘post-exertion neurogenic exhaust’ AND ‘adaptive pacing’.

‘Myalgic encephalomyelitis/chronic fatigue syndrome’ OR ‘Myalgic encephalomyelitis’ OR ‘chronic fatigue syndrome’ OR ‘PEM’ OR ‘post exertional malaise’ OR ‘pene’ OR ‘post-exertion neurogenic exhaust’ AND ‘pacing’

‘Myalgic encephalomyelitis/chronic fatigue syndrome’ OR ‘Myalgic encephalomyelitis’ OR ‘CFS’ OR ‘chronic fatigue syndrome’ OR ‘PEM’ OR ‘post exertional malaise’ OR ‘pene’ OR ‘post-exertion neurogenic exhaust’ AND ‘adaptive pacing’.
